# Annual of the Universal Medical Sciences

**Published:** 1892-12

**Authors:** 


*Annual of the Universal Medical Sciences.* Edited by CHARLES E. SAJOUS, M.D. Five volumes. Philadelphia: F. A. DAVIS. 1892.

Dr. Sajous and his staff may again be congratulated on having brought this year's efforts to the successful issue we see before us. The work is a gigantic one, and is year by year becoming more valuable and better known. Now in its fifth year, it has obtained a position which may well be envied, and from which any untimely downfall is scarcely possible. The world cannot but be grateful for such a work as this, which has rendered itself indispensable to all medical men who wish to acquire or maintain any position amongst their fellows.

